# Processing incomplete questionnaire data into continuous digital biomarkers for addiction monitoring

**DOI:** 10.1371/journal.pone.0271465

**Published:** 2022-07-14

**Authors:** Andreas Zetterström, Gunnar Dahlberg, Sara Lundqvist, Markku D. Hämäläinen, Maria Winkvist, Fred Nyberg, Karl Andersson

**Affiliations:** 1 Kontigo Care AB, Uppsala, Sweden; 2 Department of Pharmaceutical Biosciences, Uppsala University, Uppsala, Sweden; 3 Department of Immunology, Rudbeck Laboratory, Genetics and Pathology, Uppsala University, Uppsala, Sweden; 4 Ridgeview Instruments AB, Uppsala, Sweden; Mayo Clinic College of Medicine, UNITED STATES

## Abstract

**Purpose:**

eHealth systems allow efficient daily smartphone-based collection of self-reported data on mood, wellbeing, routines, and motivation; however, missing data is frequent. Within addictive disorders, missing data may reflect lack of motivation to stay sober. We hypothesize that qualitative questionnaire data contains valuable information, which after proper handling of missing data becomes more useful for practitioners.

**Methods:**

Anonymized data from daily questionnaires containing 11 questions was collected with an eHealth system for 751 patients with alcohol use disorder (AUD). Two digital continuous biomarkers were composed from 9 wellbeing questions (WeBe-i) and from two questions representing motivation/self-confidence to remain sober (MotSC-i). To investigate possible loss of information in the process of composing the digital biomarkers, performance of neural networks to predict exacerbation events (relapse) in alcohol use disorder was compared.

**Results:**

Long short-term memory (LSTM) neural networks predicted a coming exacerbation event 1–3 days (AUC 0.68–0.70) and 5–7 days (AUC 0.65–0.68) in advance on unseen patients. The predictive capability of digital biomarkers and raw questionnaire data was equal, indicating no loss of information. The transformation into digital biomarkers enable a continuous graphical display of each patient’s clinical course and a combined interpretation of qualitative and quantitative aspects of recovery on a time scale.

**Conclusion:**

By transforming questionnaire data with large proportion of missing data into continuous digital biomarkers, the information captured by questionnaires can be more easily used in clinical practice. Information, assessed by the capability to predict exacerbation events of AUD, is preserved when processing raw questionnaire data into digital biomarkers.

## Introduction

Questionnaires are widely used to diagnose addiction and other psychiatric disorders. The complexity of questionnaires varies widely, not only in the questions asked but also in number of questions, complexity, length of text and available answering alternatives. Well-known problems with questionnaire data quality include lack of compliance, subjectivity, lack of recall at time of conducting and time resolution. Digital questionnaires (e.g. Ecological momentary assessment (EMA)) solve some of these known problems, particularly recall and time resolution [1, 2], but data interpretation can still be difficult due to limitations related to measurement reactivity, acceptability, over-reporting of cravings, under-reporting of substance use, poor compliance and issues with non-random missing data [3–5]. For daily monitoring, the number of questions and the amount and complexity of text should be adjusted to not become a usability burden, but even then, daily monitoring is demanding for the subject–resulting in high frequency of days with complete lack of compliance, i.e. with all data missing.

Underlying factors thought to cause relapse in addictive disorders have been investigated and predictor variables include background data (social background, age, sex, negative life events) and self-reported data on cravings, coping resources, affective and other mood parameters [6, 7]. Recent development of EMA [2, 8] has made it possible to frequently electronically report cravings, mood and substance use. This kind of data has been used in correlation/forecasting studies and in all of these studies, the outcome measure has been self-reported substance use [4, 9–11].

Use of questionnaires for the purpose of predicting relapse has been evaluated in the past with poor success. The Addiction-Comprehensive Health Enhancement Support System (AChess) used self-reported alcohol/drug use from weekly collected questionnaires as response (y) and historical lapses/relapses combined with 10 questions from weekly questionnaire monitoring the recovery process (cravings, sleep, AA-meetings, routines, etc) as input (x) in predictive modelling [10]. When quantifying the recovery progress, lapse history data accounted for about 20% of the variance whereas the questionnaire section only accounted for ~3%. Scott et al [9] found that the relapse history dominated the predictive capability of EMA, and that there were strong interaction effects between substance use and negative affect and craving behaviour.

Addictive disorders have a unique relationship to missing data. This concerns information, as recently studied for AUD [12–15]. The meaning of missing data affects the method of representing a missing data point, known as imputation strategy. Different imputation strategies are usually tested and the one giving best predictions is selected [16]; however, missing data as such is not considered as information.

For a caregiver, it is difficult to detect and interpret data that is lacking. When raw questionnaire data is used, it is easy to interpret information collected a day when the patient has answered questions. However, it is obviously far more problematic to interpret the wellbeing or motivation state of a patient who has not answered the questions. Moreover, question answers that were given by a patient 1, 3, or 5 days ago or even longer back in time do not necessary give the caregiver correct information about the current state. A good state 3 days earlier does not imply that this state remains 3 days later. It is fair to believe that failure to comply with the daily answering routine constitutes a warning sign and implies a worsening state. Awareness of the problem with sparse answering and large quantities of missing data for addictive disorders led us to develop a construct serving as a digital biomarker, Addiction Monitoring Index (AMI). This construct combines the information in results from performed and pattern in omitted breathalyzer-based sobriety tests [12]. For alcohol use, AMI has been validated by correlation with phosphatidyl ethanol which is a 100% selective blood biomarker of alcohol use and the golden standard in Swedish medical treatment of AUD. This verifies that omission of breathalyzer tests by the patient is often explained by actual “secret drinking” [12–15]. Caregivers using our current eHealth system have been in contact with us and requested similar digital biomarkers for wellbeing and motivation as for alcohol sobriety, with imputed values for periods with missing data.

This paper proposes and discusses a method for turning frequently collected qualitative questionnaire data, where large quantities of missing data are expected, into usable continuous predictive variables without missing data. It represents a mixed-mode method for turning irregular qualitative data into a continuous actionable piece of information for the care provider to use in practice.

## Methods

The 11 questions used for daily monitoring of patients with addictive disorders were selected by a panel of care providers (Table 1) representing known positive lifestyle and risk factors for relapse in alcohol use disorder. The answers were coded into numeric values in the range from 0–100, where a high value reflects a positive outcome.

**Table 1:** Questionnaire input table and the numeric coding of the answers.

Question	Value in database					Abbreviation
	0	25	50	75	100	
How was your day?	Very bad	Bad	So-so	Good	Very good	HowWas
How did you sleep?	Very badly	Badly	So-so	Well	Very well	Sleep
How did you eat?	Very badly	Badly	So-so	Well	Very well	Eat
How was school/internship/training/work?	Very bad	Bad	So-so	Good	Very good	WorkSchool
How well did you follow your plans?	Very badly	Badly	So-so	Well	Very well	Routine
Have you done any exercise?	No	-	In my everyday life	-	Yes	Exercise
Have you socialized with anyone?	No	-	Just shortly	-	Yes	Socialized
Have anyone made you angry, sad or irritated?	Lots of people	-	A few people	-	No one	Angry
Have you felt stressed?	Yes, very much so	Yes, quite a bit	Yes, a little	Not so much	Not at all	Stress
How motivated are you to stay sober today?	Not at all	Rather unmotivated	A little motivated	Strongly motivated	Very strongly motivated	Motivation
Do you trust yourself to stay sober today?	Not at all	Not so much	I think so	Yes, I do	Yes, without any doubt	SelfConf

The database contained 751 patients who in total had answered the 11 questions (Table 2) between 31 144 and 72 215 times. The majority of patients attended therapies based on a) motivational enhancement, b) cognitive behavioral, and c) 12-step facilitation which are the therapies typically used in Swedish municipalities for treatment of patients with AUD. Due to anonymization, no information about gender, age and similar was part of the analyzed data set. Complete data (answers to all 11 questions) was available for 274 patients with a total of 23 690 patient days.

**Table 2:** Summary of the questionnaire data (row 1–11), average data (12–13) and the imputed digital biomarkers (14–15). The imputation increases the number of days (N) with available data with a factor of ~2.

	N	Mean	Std Dev	Median
HowWasDay	55 399	73.3	21.2	75
Sleep	72 215	68.6	25.0	75
Eat	51 328	71.4	20.5	75
WorkSchool	31 144	73.6	23.4	75
Routine	52 155	79.2	21.0	75
Exercise	42 613	54.4	35.8	50
Socialized	46 200	76.0	38.4	100
Angry	50 647	91.1	20.4	100
Stress	54 676	76.9	26.9	75
Motivation	66 854	89.1	17.6	100
SelfConf	46 494	90.6	17.3	100
WeBe	76 624	75.1	18.3	75
MotSC	68 418	89.8	16.6	100
WeBe-i	130 096	50.4	31.6	57.5
MotSC-i	118 335	59.5	37.0	71.4

### Creation of digital biomarkers WeBe-i and MotSC-i

A generic description of the algorithm that was used to create the digital biomarkers WeBe-i and MotSC-i is shown in Table 3. In a first step of designing digital biomarkers, the variance structure of the raw input data was analyzed using Principal component analysis (PCA) on complete patient days (i.e. with all questions answered) only. The PCA model were varimax rotated into factors and the most contributing parameters per factor were identified. The average of the contributing questions was calculated to provide the foundation of two different digital biomarkers. For missing data, an imputation strategy was defined (Table 3).

**Table 3:** Generic algorithm for constructing a questionnaire based digital biomarker.

Step 1: Calculate raw daily value. For a day with at least one answer to at least one question part of the digital biomarker, calculate the average of all available answers. Define **raw daily value** as the calculated average. For a day with no answer to any question part of the digital biomarker, impute the **raw daily value** according to the following: Find the last day before the current day, for which questions have been answered (“last answered day”) Count the number of days passed since the last answered day If the number of days passed since the last answered day is 1, set the raw daily value to (2/3 * [the raw daily value for the last answered day]) If the number of days passed since the last answered day is 2, set the raw daily value to (1/3 * [the raw daily value for the last answered day]) If the number of days passed since the last answered day is > = 3, set the raw daily value to 0
Step 2: Construct digital biomarker. Create the value of the digital biomarker (S) for day t as an exponentially smoothened value calculated from the daily raw values (x) over time. S_0_ = x_0_ S_t_ = αx_t_ + (1 –α) * S_t-1_, t > 0 Where α is the smoothing factor, and 0<α<1.

In a second step, the value of a digital biomarker at any given point in time was calculated as an exponentially smoothened average of recent average questionnaire results (actual or imputed). This produces both the ability to extract a biomarker value at any point in time and a seemingly continuous biomarker value.

The underlying hypothesis behind the imputation of raw daily values for days without questionnaire answers is that failure to comply with tasks correlates with a worsening state. This has been used in the past for AUD (12, 15). The use of exponential smoothening implicitly means that an answer has a practical impact on the digital biomarker value in the coming days (weighted by a smoothing factor), and that wellbeing and motivation are measured from a more long-term perspective than just a single-point-in-time value.

### LSTM networks

A Long Short-term Memory (LSTM) network is a neural network [17] that processes data given to it in one end using weights (internal numbers that constitute the network’s knowledge) and in the other end outputs an “answer” or a “prediction”. By comparing the output to a pre-labelled “ground truth”, and using mathematical functions to update the weights accordingly, it can learn from the training data by minimizing a loss function. What distinguishes an LSTM neural network is that its architecture encompasses a time-step dimension which makes it aware of the temporal structure of the data, and thus is particularly adapted to time-series data [18].

### Forecasting studies

To evaluate possible information loss in the process of generating digital biomarkers from questionnaire data, the digital biomarkers were compared to raw questionnaire data with respect to their power to predict exacerbation events (EE), as measured by eHealth scheduled breathalyzer test results and test compliance. LSTM neural networks were trained to predict an exacerbation event of length > = 2 days. An EE represents a rapid worsening of the disease state [15]. This analysis served two purposes: 1) determine whether there is information loss when averaging/imputing/exponentially smoothing the raw question answer values, and 2) determine, more generally, whether wellbeing/motivation question answer data in any way can be used to predict the clinical course of AUD. Two different look-ahead ranges were used: 1–3 days and 5–7 days ahead. The output from the LSTM networks was a prediction of the probability of an EE of length > = 2 days starting within the respective look-ahead range in the future. By applying a threshold value that yielded the maximum Matthews Correlation Coefficient (MCC) the probability prediction was converted into a binary true/false prediction. The predictive power was assessed using the Area under the ROC curve (AUC) for the probability prediction and Sensitivity/Specificity/MCC. Three different LSTM networks were trained, each using different input data. Input data for each day for the different networks were:

1. Raw question answer values for the 11 questions + true/false whether the patient answered the particular question on that day (22 features in total)
2. Average of raw question answer values for Motivation/Self-Confidence questions, true/false whether any motivation question was answered on that day, average of raw question answer values for Wellbeing questions and true/false whether any wellbeing question was answered on that day (4 features in total)
3. Digital biomarker MotSC-i, digital biomarker WeBe-i (2 features in total)

Data from the first 7 days of the patient’s treatment periods was excluded from the dataset (start-up period). The resulting dataset consisted of 115 641 patient days in total. The dataset was randomly split into partitions, by patient, into Training (527 patients, 84 420 patient days), Validation (used to determine when the model training was finished, 112 patients, 15 803 patient days) and Test (112 patients, 15 418 patient days). A minimum of 7 days history was provided for each day, up to a maximum history length of 93 days. The performance of the LSTM network was evaluated on the Test dataset. For evaluation, prediction was issued by the networks for each day when the patient was not already in an EE and the AMI was > = 40. It should be noted that all LSTM networks had the same hyperparameters and they were not tuned to best suit the respective input data.

## Results

**Fig 1:**
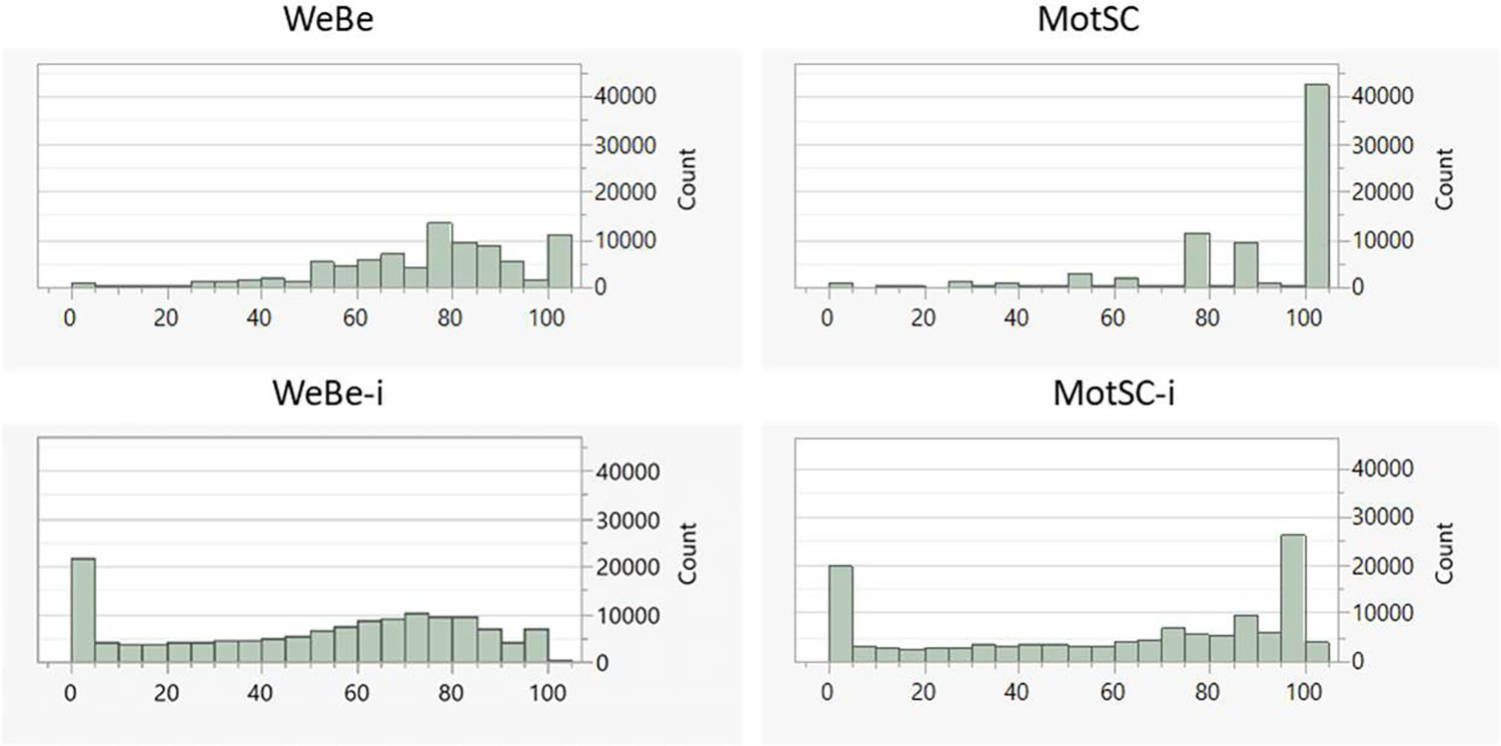
Distribution of averaged wellbeing data, WeBe, and motivation and self-confidence data, MotSC, (top) and the corresponding digital biomarkers, WeBe-i and MotSC-i (bottom).

The analysis of complete questionnaire data (n = 23 690 patient days, 274 patients) resulted in three strong principal components with eigenvalues larger than 1 (PC1-3) which explained 59.7% of the variance in data. After varimax rotation and discussion with health care providers, dominant contributors to factor 1 and 3 were combined into one digital biomarker WeBe-i (9 questions; related to wellbeing) and dominant contributors to factor 2 were combined into one digital biomarker MotSC-i (2 questions; related to motivation and self-confidence to stay sober), see Table 4. A smoothing factor of 0.32 was used for both digital biomarkers. After removal of inactive tails and transforming data into digital biomarkers (according to full use of Table 3), 130 096 WeBe-i and 118 335 MotSC-i daily values were obtained. Thus, digital biomarkers provided actionable data for a patient day up to 5.4 times more often than did completely answered questionnaires. The effects of transforming raw questionnaire data into digital biomarkers are exemplified in Fig 1. The effect of the exponential transformation smooths the data and gives a large increase in number of low values in WeBe-i and MotSC-i data (and in clearly lower mean index values, Table 2). This reflects that many patients have long periods with missing data.

**Table 4:** Principal components (PC1-3), varimax rotated factor loadings and the belonging to the 2 digital biomarkers.

	PC1	PC2	PC3	Factor 1	Factor 2	Factor 3	WeBe-i	MotSC-i
HowWas	0.82	0.11	-0.15	0.79	0.19	0.21	x	
Stress	0.73	-0.19	-0.21	0.72	0.27	-0.09	x	
Eat	0.70	0.17	-0.24	0.74	0.04	0.18	x	
Routine	0.70	0.04	-0.02	0.62	0.27	0.19	x	
WorkSchool	0.70	0.07	-0.12	0.67	0.17	0.16	x	
Sleep	0.69	0.14	-0.22	0.71	0.06	0.17	x	
Motivation	0.61	-0.40	0.51	0.26	0.85	0.08		x
Socialized	0.32	0.57	0.38	0.12	0.09	0.74	x	
SelfConf	0.53	-0.44	0.59	0.15	0.89	0.06		x
Angry	0.42	-0.34	-0.38	0.54	0.10	-0.37	x	
Exercise	0.35	0.56	0.30	0.19	0.05	0.70	x	
Explained variation (%)	38.4	10.9	10.7					
Eigenvalue	4.23	1.20	1.17					

In comparison, when averaging raw questionnaire data from contributing factors (Table 3, only conducting averaging on available data in step 1) to WeBe-i and MotSC-i each day, 76 624 (WeBe factors) and 68 418 (MotSC factors) days of averaged data were obtained. Thus digital biomarkers provided actionable data nearly twice as often than days where at least one answer to the contributing questions for the respective digital biomarkers was available.

**Fig 2:**
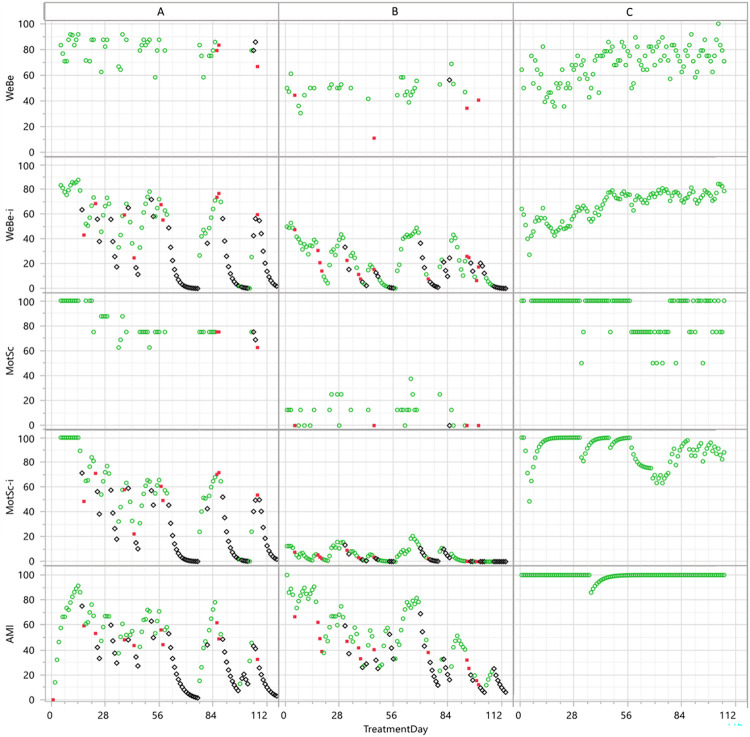
Depicting the clinical course of patients using wellbeing and motivation/self-confidence data. The average/digital biomarker view of wellbeing (WeBe/WeBe-i), motivation/self-confidence (MotSc/MotSc-i), and Addiction Monitoring Index (AMI) data for 3 patients (A-C) as time series during 4 months (x-axis = Treatment day). Symbols: Green circle = no alcohol detected; Red square = alcohol detected; Black diamond = all breathalyzer tests omitted.

The effect of transforming average data (WeBe/MotSc) into digital biomarkers (WeBe-i/MotSc-i) is depicted in Fig 2 using a 16 week time series of 3 patients. Patient A/B/C answered 40/30/97% of the days, i.e. more than half of the questionnaire data was missing for patient A and B. The average WeBe/MotSc data for patient A (80/83) and B (47/11) differ in average levels but is quite stable over time. However, both have several gaps, some shorter and some 1–3 week long, with missing data (Fig 2, black diamonds in WeBe-i and MotSC-i graphs) and alcohol was detected 10/13 days (red squares). This is captured in the WeBe-i and MotSc-i time series where the detected alcohol (red squares) in the beginning and omitted breathalyzer test (black diamonds) during exacerbation events are captured in the decreasing index values (Fig 2). The individual and qualitative aspect of questionnaire data is also evident when comparing the timeseries of the 3 patients. Patient A and B have similar average clinical course based on detected alcohol and breathalyzer test compliance (Fig 2. AMI) but they drastically differ in both WeBe (80/47) and MotSc (83/11) levels, i.e. there is no direct linear relationship with drinking. Patient A and C have similar average WeBe (80/69) and MotSC (83/91) whilst their WeBe-i (43/67) and MotSC-i (44/89) differ considerably due to answering compliance, which is also reflected in sobriety (alcohol detected 10/0 times).

### Rationale for refining raw data into digital biomarkers

The impact of transforming questionnaire data into digital biomarkers through imputation was evaluated as the capability of predicting exacerbation events using LSTM neural networks. Results for the three different LSTM network models are displayed in Table 5. There is predictive power to forecast a future exacerbation event in wellbeing/motivation question answer data both 1–3 days in the future (Table 5 section A) and 5–7 days in the future (Table 5 section B). It further appears that information does not seem to be lost–and/or no erroneous information seems to be added–when averaging, imputing missing answers and exponentially smoothing the data into the digital biomarkers. The minor perceived gain in predictive power (slightly higher MCC for row 3 and 6) by refining the raw data into digital biomarkers is not significant.

**Table 5:** The performance of the LSTM neural network model’s capability to predict exacerbation events (EE) of unseen patients from the Test dataset. True Positives (TP), True Negatives (TN), False Positives (FP), False Negatives (FN), Sensitivity, Specificity and Matthews Correlation Coefficient (MCC) were calculated using the threshold that yielded the maximum MCC. Section A refers to predicting EEs occurring 1–3 days in the future, and section B refers to predicting EEs occurring 5–7 days in the future.

Input data	N input features	Look-ahead range (days)	N patients (Test set)	N patient days	TP	TN	FP	FN	AUC	Sensitivity	Specificity	MCC
Section A												
Raw question *^1^ answer data	22	1–3	112	15 418	830	4507	5174	153	0.686	0.844	0.466	0.181
Average of MotSC/WeBe factors*^2^	4	1–3	112	15 418	812	4670	5011	171	0.683	0.826	0.482	0.179
Digital biomarkers: MotSC-i/WeBe-i	2	1–3	112	15 418	748	5775	3906	235	0.699	0.761	0.597	0.209
Section B												
Raw question *^1^ answer data	22	5–7	112	15 418	765	5082	4599	218	0.649	0.778	0.495	0.175
Average of MotSC/WeBe factors*^2^	4	5–7	112	15 418	764	4793	4888	219	0.649	0.788	0.525	0.158
Digital biomarkers: MotSC-i/WeBe-i	2	5–7	112	15 418	773	5169	4512	210	0.681	0.786	0.534	0.185

*1. Answers to the 11 questions and a 1/0 whether an answer is missing for each question.

*2. The 2 average values and a 1/0 whether the respective average values exist for the day or not.

## Discussion

In this paper we show that questionnaire data with massive amounts of missing data may contain valuable information. To access the available information, transformation of questionnaire data into digital biomarkers is beneficial. The method discussed in this paper is one possible route for providing accurate, daily, and continuously graded access to the collected irregular, qualitative questionnaire information. It is possible that by applying similar data transformation in other questionnaire-based research fields, the value of questionnaire data may increase significantly in cases where the missing data is information in itself.

The need to have a daily composite value on wellbeing and motivation/self-confidence, instead of occasionally appearing missing data, was suggested by the eHealth system users who routinely use the digital biomarker AMI for daily monitoring of sobriety and compliance of patients with AUD. In AUD, the value of missing data is unique, the individual will typically lose motivation to engage in whatever activity (including answering questionnaires) when approaching an exacerbation event. Thus, an imputation strategy must be carefully constructed to capture the essence of missing data. In the case of AUD, an individual that omits questionnaire responses causing missing data may be approaching exacerbation but may also be unavailable or just forgetful. Caution and balance are strongly recommended when designing imputation strategy, to not introduce unnecessary bias.

Which type of data is the collected wellbeing and motivation/self-confidence data? The fact that there is 3–5 answering alternatives with a gradual scale might give the view of that the data is quantitative or at least ordinal. However, the interpretation of “Yes without any doubt” and the use of a gradual subjective Likert type of scale may vary considerably between different persons. The use of scale values also differs between cultures and nations [19, 20]. Hence, a gradual scale sometimes must be interpreted qualitatively and/or in an individualized fashion. To help the care provider to interpret the time series of incomplete questionnaire data in such an individualized manner, graphical visualization of the continuous digital biomarkers is advantageous. The time series for each patient presents an individualized view of their clinical course which makes it possible to display the individual-ordinal time series of the patients and qualitatively interpret them in relation to their historic profile. The smoothed continuous data facilitate in the identification of exacerbation events and overall time trends, e.g., the positive WeBe(-i) trend for patient C (Fig 2). A further advantage is that additional information can be displayed in the graphical overview of the WeBe-i/MotSC-i time series as exemplified in Fig 2 where the symbol shows if sobriety data is missing or if alcohol has been detected.

The data set under analysis in this paper illustrates the effect of introducing digital biomarkers. The number of days for which motivation and wellbeing could be communicated to a care provider increased immensely. Of the about 23 000 days with a complete questionnaire result, about 70 000 daily values could be generated by averaging all answers provided in a single day (meaning that one answer was sufficient to count as a reported day) to about 155 000 daily values when transformed into a digital biomarker (Table 3). This is a practical result of the imputation where missing data becomes part of the digital biomarker. There always exists a digital biomarker value available representing the current level of wellbeing, motivation and self-confidence–something that brings value to practitioners. The appearance of a large number of index values close to zero when average data is imputed is an effect of long relapse periods.

The results presented in Table 5 provide, in our opinion, a solid foundation to conclude that processing the data into digital biomarkers does not imply a significant loss of information or introduction of erroneous information. The exponential smoothing introduces the concept of historic baggage, which is intuitive but whose rationale is not obvious. The fact that the predictive power is not diminished for the biomarkers also provides a foundation for our conclusion that the historic baggage concept might be suitable also to wellbeing/motivation question answer data, just as it has been shown to be suitable for monitoring the clinical course of AUD (in the form of the digital biomarker AMI, [12]). Hence this implies that the digital biomarkers are accurate representations of the questionnaire data. We also tested prediction using 5–7 days before onset of an EE with comparable results as using 1–3 days.

There are two aspects of this work where the decisions were of ad hoc type. The first is the selection of 0.32 as α, i.e. the influence of the last day on the exponentially smoothed digital biomarkers (WeBe-i, MotSC-i; Table 3). In our initial testing of different α we found that using 0.21 (as previously, [12]) yielded only significant dips in WeBe-i/MotSC-i when data was missing (i.e. did not reflect variation in answers). Higher alphas we tested by overly plotting of data series from individual patients and compared with AMI. An α = 0.32 was selected to make the digital biomarkers (WeBe-i and MotSC-i) as closely as possible resemble the pattern of AMI. The second aspect is to use only two digital biomarkers for data compression even though the principal component model contained three factors with clear contribution (eigenvalues larger than 1). The decision to combine motivation and self-confidence into one factor was supported by varimax rotated factor analysis. It is also well known from the past that high motivation and self-confidence to stay sober are important factors to remain sober [21]. The decision to combine the remaining 9 variables into 1 factor was based on the following: a) PC1 contained a significant contribution from all these 9 variables. b) The rotated third factor was composed of Socialized/Exercised/(-Angry), i.e. the only questions with only 3 alternatives as answers, a fact that may be the underlying reason for principal component analysis to separate these three from the set of 9.

We show that answers to wellbeing and questionnaire answers contain useful information to forecast the clinical course of AUD. The questionnaire contains several questions, each weakly contributing to the estimated risk of relapse. When combining these several weakly contributing pieces of information, supplementing with compliance to tasks (measured through imputing missed test as a risk factor), and incorporating historic answers through exponential smoothing, continuous (both in time and magnitude) digital biomarkers are achieved. Hence, digital biomarkers from questionnaires can provide healthcare providers with a condensed daily view of patient status, in our example wellbeing, motivation and self-confidence, even in cases where the patient fails to comply. Prediction of whether a patient in treatment for AUD will drink the coming days allows early intervention by healthcare or family. Although the performance displayed in Table 5 for predicting upcoming exacerbation events using questionnaire-based digital biomarkers is moderate, every single exacerbation event that is prevented means improved quality of life for the patients and their families, as well as reducing care costs, which implies considerable cost savings for society. The prediction models displayed in Table 5 would however need improvement prior to use in any clinical setting. That said, since the digital biomarkers provide a daily condensed view of wellbeing and motivation, the health care provider has a tool to intervene at an early stage, even in cases where predictive forecasting models have not been developed.

## Conclusions

Management of missing data in questionnaires with an exponentially smoothed penalty for increased number of sequential days without answers seems to be suitable for wellbeing and motivation questionnaire data. The imputation strategy increases the usability of questionnaire data with large quantities of missing answers in clinical practice. Digital biomarkers related to wellbeing and motivation can be provided continuously to care practitioners and could bring power for predicting the clinical course of patients with alcohol use disorder.

## Supporting information

S1 Dataset (ZIP)
